# Supplementary material for: Association of Financial Distress and Monthly Income with Smoking During the COVID-19 Pandemic Recession in Thailand: A Nationwide Cross-Sectional Study
Source: Int J Environ Res Public Health. 2025 Aug 18;22(8):1287. doi: 10.3390/ijerph22081287 (PMC12386154; doi:10.3390/ijerph22081287)
Supplement: Supplementary file 1 [file ijerph-22-01287-s001.zip › ijerph-3741537-supplementary.pdf]

ID _ _ _ _

Questionnaire
Survey of tobacco product use during the COVID-19 epidemic Wave 3
(answered during June 2021) in 4 regions of Thailand
No more than 15 minutes of time required to answer

Data collector .... Date questionnaire completed ....
Health Region .... Province .... District ....
Area [ ] 1) Urban [ ] 2) Rural

Section 1. General Information

**1. Gender**		
	[ ] 1) Male	[ ] 2) Female
**2. Age** .... Years (please specify)		
**3. Monthly income** .... Thai Bahts (please specify)		
**4. Number of household members** .... persons (please specify)		
**5. Work during the COVID-19 epidemic, Wave 3**		
[ ] 1) Came to work as normal	[ ] 2) Bring work to do at home. Days per week	[ ] 3) Number of working days was reduced
[ ] 4) Temporary suspension of employment	[ ] 5) Termination of employment or business closed	[ ] 6) Was let go from work
[ ] 7) Employment contract expired	[ ] 8) Resignation	[ ] 9) Others

Section 2. Tobacco consumption behavior (normal / during Wave 3 of the COVID-19 Epidemic)

**6. Do you currently smoke cigarettes/tobacco products during the COVID-19 Epidemic, Wave 3?**		
[ ] Yes (answer Question 7 next)	[ ] No (answer Section 5)	[ ] Yes and I have quit<br>Quit for .... days<br>Quit for .... months<br>(choose one answer, then go answer Section 5)

**7. Before the COVID-19 epidemic, during normal situation, you smoked** .... cigarettes **per day** (please specify)
**8. During the COVID-19 epidemic, Wave 3, you smoked** .... cigarettes **per day** (please specify)
**9. What is your smoking behavior during the COVID-19 epidemic, Wave 3?**

[ ] Smoke the same amount (answer Question 10)	[ ] Smoke more (answer Question 11)	[ ] Smoke less (answer Question 12)	[ ] Stopped consuming tobacco products (answer Question 13)

**10. Reason(s) for consuming tobacco products <u>in the same amount</u> during the COVID-19 epidemic, Wave 3 (can choose more than 1 response)**	
[ ] Cannot quit	[ ] Not worried about the COVID-19 situation
[ ] Stressed from termination of employment/reduction of salary	[ ] Don't believe that smoking increases the risk of COVID-19 infection
[ ] Hoarding goods / Fear of scarcity / Concerned about price	[ ] Others .... (please specify)
**11. Reason(s) for consuming tobacco products <u>in greater amounts</u> during the COVID-19 epidemic, Wave 3 (can choose more than 1 response)**	
[ ] Stressed from work	[ ] Stressed about the COVID-19 situation
[ ] Stressed from termination of employment/reduction of salary	[ ] Don't believe that smoking increases the risk of COVID-19 infection
[ ] Hoarding goods / Fear of scarcity / Concerned about price	[ ] Others .... (please specify)
**12. Reason(s) for consuming tobacco products <u>in lesser amounts</u> during the COVID-19 epidemic, Wave 3 (can choose more than 1 response)**	
[ ] Reduced income	[ ] Increased expenses
[ ] It's harder to find and buy tobacco products	[ ] Wanting to care for my own health
[ ] Concerned that smoking increases the risk of COVID-19 infection	[ ] Concerned about the effect on my family members' health
[ ] Others .... (please specify)	
**13. Reason(s) for <u>stopping the consumption of tobacco products</u> during the COVID-19 epidemic, Wave 3 (can choose more than 1 response)**	
[ ] Reduced income	[ ] Increased expenses
[ ] It's harder to find and buy tobacco products	[ ] Wanting to care for my own health
[ ] Concerned that smoking increases the risk of COVID-19 infection	[ ] Concerned about the effect on my family members' health
[ ] Others .... (please specify)	

Section 3. Type of tobacco product used during normal situation / COVID-19 epidemic Wave 3

**14. Type of tobacco product used before the COVID-19 epidemic (more than 1 answers allowed)**		
[ ] Hand-rolled loose-leaf tobacco	[ ] Thai brands cigarettes	[ ] Foreign brands cigarettes
[ ] Electronic cigarette	[ ] Cigars	[ ] Hookah / Baraku
[ ] Cigarettes priced below market values	[ ] Others	
**15. In case of cigarettes, which flavor did you use before the epidemic?**		
[ ] Regular (no cooling)	[ ] Menthol (cooling)	
[ ] Flavored (fruity scents with cooling effect)		
**16. Type of tobacco product used during the COVID-19 epidemic Wave 3? (more than 1 answers allowed)**		
[ ] Hand-rolled loose-leaf tobacco	[ ] Thai brands cigarettes	[ ] Foreign brands cigarettes
[ ] Electronic cigarette	[ ] Cigars	[ ] Hookah / Baraku
[ ] Cigarettes priced below market values	[ ] Others	
**17. In case of cigarettes, which flavor did you use during the COVID-19 epidemic Wave 3?**		
[ ] Regular (no cooling)	[ ] Menthol (cooling)	
[ ] Flavored (fruity scents with cooling effect)		

Section 4. Expenses on cigarettes / other tobacco products

**18. How much did you spend per week on tobacco products during normal situations before the COVID-19 epidemic?**		
[ ] Less than 60 Bahts	[ ] 60-200 Bahts	[ ] 200 - 500 Bahts
[ ] 500 - 800 Bahts	[ ] 800 - 1,000 Bahts	[ ] More than 1,000 Bahts

**19. How much did you spend per week on tobacco products during the COVID-19 epidemic Wave 3?**		
[ ] Less than 60 Bahts	[ ] 60-200 Bahts	[ ] 200 - 500 Bahts
[ ] 500 - 800 Bahts	[ ] 800 - 1,000 Bahts	[ ] More than 1,000 Bahts

Section 5. Knowledge on the impact of tobacco product use on risk of the coronavirus (COVID-19) epidemic

**19. Smoking any type of tobacco product contributes to coronavirus (COVID-19) infection as it increases opportunities for the hand to touch the face, nose, mouth, and eyes**		
[ ] Yes	[ ] No	[ ] Not sure
**20. Regular smokers of tobacco products are at high risk of having severe lung infection from coronavirus disease**		
[ ] Yes	[ ] No	[ ] Not sure
**21. Smokers of tobacco products with coronavirus infection can spread the disease through coughing, sneezing, or breathing on others, as well as sharing the same stick of tobacco product or paraphernalia**		
[ ] Yes	[ ] No	[ ] Not sure

Section 6. The desire to quit / remain users of tobacco products, and factors for quitting / continuing

**22. Do you wish to quit using tobacco products?**	
[ ] Yes (go to Question 24)	[ ] No (go to Question 23)

**23. Why do you NOT wish to quit using tobacco products? (more than 1 answer allowed)**		
[ ] There is no effect on the body at present	[ ] Stress relief	[ ] Socialization
[ ] Feel that it's hard to quit	[ ] Others .... (please specify)	

24. Why do you wish to quit using tobacco products? (more than 1 answer allowed)		
[ ] Desire to care for health	[ ] Reduced income	[ ] Increased expenses
[ ] It's harder to find tobacco products to buy	[ ] Concerned that smoking increases risk of COVID-19 infection	[ ] Others .... (please specify)

22. Could you access smoking/tobacco cessation service systems?	
[ ] Could access services e.g., smoking cessation clinics or quite[sic] line, etc.	[ ] Could not access data / system for smoking cessation
